# Introduction to Michael E. Robbins Memorial Issue

**DOI:** 10.3109/09553002.2014.939778

**Published:** 2014-08-11

**Authors:** Dana Greene-Schloessor, Jacqueline P. Williams

**Affiliations:** ^a^ Department of Radiation Oncology, Wake Forest School of Medicine, Winston-Salem, NC; ^b^ Departments of Environmental Medicine and Radiation Oncology, University of Rochester Medical Center, Rochester, NY, USA

## Michael (Mike) E. Robbins (1954–2012)

By the time of his death on 23 November 2012, Michael (Mike) E. Robbins had gained a national and, indeed, international, reputation as a leader in the field of radiation-induced normal tissue effects. For the final decade or so of his career, Mike had been based at Wake Forest Medical Center, an institution where he had developed a world-class research program looking at the prevention and treatment of radiation-associated tissue injury, with a specific interest in radiation-induced brain injury. Although the impact of Mike's body of work in the latter area of research is clearly highlighted in the paper proffered by Prasanna et al. (2014), his death at a relatively young age cut short a career and sphere of influence that had spanned decades and continents.

Born in London, UK, in 1954, Mike began his academic career at Thames Polytechnic, London (now the University of Greenwich), and received his bachelor's degree in Applied Biology in 1976. He remained at Thames Polytechnic for his postgraduate studies, investigating the effects of parathyroid hormones on the kidney in domestic fowl, and obtained his PhD in 1980. That same year, Mike joined the staff of the Churchill Hospital Research Institute, University of Oxford, as a post-doctoral fellow, where he was first introduced to the world of radiation biology. He readily integrated his prior knowledge of kidney function with the tenets of radiation biology and, in parallel with prestigious research groups such as Moulder et al. (2014), helped to provide the foundation of work performed in renal radiation biology that subsequent investigators, such as Scharpfenecker (de Cortie et al. 2014), continue to build upon to this day. But in addition to his work on kidney, it was during this period that Mike acquired a wider reputation as an innovative researcher, leading to an appointment as the Deputy Director of the Cancer Research Campaign Normal Tissue Radiobiology Research Group, University of Oxford, from 1987–1993. In this position, Mike broadened his research scope to more diverse aspects of radiation-induced normal tissue injury: Exploring effects in tissues other than kidney, such as skin and the central nervous system; modeling clinical translational problems, an area of study that continues to be of interest to his earlier mentor and colleagues at Oxford (Jones and Hopewell 2014); and investigating tissue-organ interactions, marking the start of his interest in tissue microenvironment. This latter field of research remains relatively unexplored in respect to normal tissues, but the implications of such interactions are highlighted in the paper by the Medhora group (Gao et al. 2014).

In 1993, Mike moved to the United States, where his initial appointments were as Associate (subsequently full) Professor in the Department of Radiology and the Director of Research for the Free Radical and Radiation Biology Program at the University of Iowa. While in Iowa, his reputation as an innovative researcher within the radiation biology and the wider scientific communities continued to grow. Although his overall interests in normal tissue injury remained relatively broad until the time of his death, it was during this period that medical problems within Mike's family, most notably with regard to his wife, Lucy, brought a deeper focus to Mike's research on brain radiation biology: For example, he began to more closely investigate the response of both brain tumor and normal tissue to radiation therapy, and how interactions between the two, dependently and independently, affect late outcomes.

With his move to the Wake Forest School of Medicine in 2001, as a Professor in the Department of Radiation Oncology and Section Head of Radiation Biology, Mike continued this work, developing a senior leadership role in the Thomas K. Hearn Brain Tumor Center of Excellence. He fostered strong collaborative ties and gained the respect of investigators from both inside and outside his home institution, for example with the Blomgren (Blomstrand et al. 2014, Bostrom et al. 2014) and Limoli groups (Acharya et al. 2014). At both Iowa and Wake Forest, Mike continued to build on his reputation as a researcher, instructor, and, importantly to him, as a teacher and mentor of junior faculty. His outstanding leadership, commitment to his trainees, and excellent teaching skills are exemplified by the normal tissue papers in this issue from those that have looked to, and benefited from, Mike for mentorship and support over the years (Greene-Schloesser et al. 2014, Hutchinson et al. 2014, Peiffer et al. 2014).

In many ways, Mike Robbins’ work speaks for itself, but does not describe the man. A sociable and loyal companion and friend, Mike was devastated by the loss of Lucy, but counted himself fortunate to subsequently meet and marry Pam (née Scordas) and, with her support, continued his scientific initiatives against cancer, up to and even during his own illness. In addition to research, Mike had other passions, including racing/sport motorcycles, World War II history, traveling, and hiking with his wife and dogs, Dash, Chase, and Stella. For many of us within the radiation biology community, Mike was a beloved friend and colleague and will be greatly missed, especially for his keen scientific mind, his passion and enthusiasm, his mentoring and counsel, and for his dry sense of humor.

Mike is survived by his wife Pam, his mother, Liliane, his brother, Mark and sister, Monique.

## References

[CIT0001] Acharya M, Martiroian V, Christie L-A, Limoli C (2014). Long-term cognitive effects of human stem cell transplantation in the irradiated brain. Int J Radiat Biol.

[CIT0002] Blomstrand M, Kalm M, Grandér R, Björk-Eriksson T, Blomgren K (2014). Different reactions to irradiation in the juvenile and adult hippocampus. Int J Radiat Biol.

[CIT0003] Bostrom M, Kalm M, Hellström Erkenstam N, Kaluza D, Jakobsson L, Kalm M, Blomgren K (2014). The hippocampal neurovascular niche during normal development and after irradiation to the juvenile mouse brain. Int J Radiat Biol.

[CIT0004] de Cortie K, Russell N, Coppes R, Stewart F, Scharpfenecker M (2014). Bone marrow-derived macrophages incorporate into the endothelium and influence vascular and renal function after irradiation. Int J Radiat Biol.

[CIT0005] Gao F, Fish B, Szabo A, Schock A, Narayana J, Jacobs E, Moulder J, Lazarova Z, Medhora M (2014). Enhanced survival from radiation pneumonitis by combined irradiation to the skin. Int J Radiat Biol.

[CIT0006] Greene-Schloesser DM, Kooshki M, Payne V, D’Agostino RB, Wheeler KT, Metheny-Barlow LJ, Robbins ME (2014). Cellular response of the rat brain to single doses of ^137^Cs γ rays does not predict its response to prolonged “biologically equivalent” fractionated doses. Int J Radiat Biol.

[CIT0007] Hutchinson ID, Olson J, Lindburg CA, Payne V, Collins B, Smith T, Munley MT, Wheeler KT, Willey JS (2014). Total-body irradiation produces late degenerative joint damage in rats. Int J Radiat Biol.

[CIT0008] Jones B, Hopewell J (2014). Alternative models for estimating the radiotherapy re-treatment dose for the spinal cord. Int J Radiat Biol.

[CIT0009] Moulder JE, Cohen EP, Fish BL (2014). Mitigation of experimental radiation nephropathy by renin-equivalent doses of angiotensin converting enzyme inhibitors. Int J Radiat Biol.

[CIT0010] Peiffer A, Creer R, Linville C, Olson J, Kulkarni P, Brown J, Riddle D, Robbins ME, Brunso-Bechtold J (2014). Radiation induced cognitive impairment and altered diffusion tensor imaging in a juvenile rat model of cranial radiotherapy. Int J Radiat Biol.

[CIT0011] Prasanna PGS, Ahmed MM, Stone HB, Vikram B, Mehta MP, Coleman CN (2014). Radiation-induced brain damage, impact of Robbins’ work and the need for predictive biomarkers. Int J Radiat Biol.

